# Diagnostics of Melanocytic Skin Tumours by a Combination of Ultrasonic, Dermatoscopic and Spectrophotometric Image Parameters

**DOI:** 10.3390/diagnostics10090632

**Published:** 2020-08-26

**Authors:** Kumar Anubhav Tiwari, Renaldas Raišutis, Jokūbas Liutkus, Skaidra Valiukevičienė

**Affiliations:** 1 Ultrasound Research Institute, Kaunas University of Technology, K. Baršausko St. 59, LT-51423 Kaunas, Lithuania; renaldas.raisutis@ktu.lt; 2 Department of Skin and Venereal Diseases, Lithuanian University of Health Sciences, Eivenių str. 2, LT-50161 Kaunas, Lithuania; jokubas@liutkus.lt (J.L.); skaidra.valiukeviciene@kaunoklinikos.lt (S.V.)

**Keywords:** melanoma, melanocytic tumor, dermatoscopy, ultrasonography, spectrophotometry, classification, diagnostics

## Abstract

Dermatoscopy, high-frequency ultrasonography (HFUS) and spectrophotometry are promising quantitative imaging techniques for the investigation and diagnostics of cutaneous melanocytic tumors. In this paper, we propose the hybrid technique and automatic prognostic models by combining the quantitative image parameters of ultrasonic B-scan images, dermatoscopic and spectrophotometric images (melanin, blood and collagen) to increase accuracy in the diagnostics of cutaneous melanoma. The extracted sets of various quantitative parameters and features of dermatoscopic, ultrasonic and spectrometric images were used to develop the four different classification models: logistic regression (LR), linear discriminant analysis (LDA), support vector machine (SVM) and Naive Bayes. The results were compared to the combination of only two techniques out of three. The reliable differentiation between melanocytic naevus and melanoma were achieved by the proposed technique. The accuracy of more than 90% was estimated in the case of LR, LDA and SVM by the proposed method.

## 1. Introduction

In Europe, cutaneous melanoma (CM) is the fifth most common type of cancer, with an incidence of 15.0 in age-standardized rate (ASR per 100,000 person-years). Northern Europe displays the largest ASR mortality of 3.8 in the region, with an incidence of 23.4 [1]. CM incidence rate shows high worldwide variability: it ranges from 0.30 in South-Central Asia to 33.6 in Australia and New Zealand [2]. The US Preventive Services Task Force does not currently recommend CM screening in the general population [3] and the introduction of such programs in Germany wielded inconclusive results [4]. A systematic review highlighted the key risk factors for CM screening initiation in high-risk individuals: a large number of melanocytic or dysplastic naevi, a family history of melanoma, light (Fitzpatrick I and II) skin types [5].

CM can be classified according to clinical and pathological features, based on the updated American Joint Committee on Cancer (AJCC) staging system [6]. Additional genetic classification is frequently used in research settings and has an expanding role in treatment selection: BRAF, Ras, NF-1, wild-type and other genetic subtypes have been identified [7]. Excision of primary tumor remains essential in diagnosing CM and includes histopathological measurement of tumor thickness according to Breslow to the nearest 0.1 mm [6,8]. Although new CM management guidelines highlight the potential uses of emerging diagnostic technologies, further research is needed to acquire evidence on efficacy and utility [9]. Currently, the decision to biopsy a skin lesion are generally based on the dermatoscopic evaluation by a specialist, which has shown superior sensitivity (0.90 vs. 0.71) and specificity (0.90 vs. 0.80) to naked-eye examination [10]. However, a meta-analysis showed that 15 pigmented lesions were required to treat (excise) for a CM diagnosis, which is a significant burden for the general populace [11]. Additional factors, such as CM transection [4], lack of standardization in tissue sectioning [12] and uncertainties in pathology report reproducibility and accuracy [13,14], and stress the potential uses of more sophisticated diagnostic methods, including modern imaging, machine learning and radiomics [15,16].

Computer-aided diagnostics (CAD) of CM are approaching the level of dermatologists, both outside and inside clinical settings [17,18]. A recent meta-analysis showed that the sensitivity and specificity of various such systems stood at 0.74 (95% CI, 0.66–0.80) and 0.84 (95% CI, 0.79–0.88), respectively [19]. Modern convolutional neural networks (CNN), currently using photographic and dermatoscopic images for testing, report even better results than naked-eye examination [20,21,22]. Accuracy can be further increased using additional data from novel non-invasive imaging systems. Commonly used technologies include reflectance confocal microscopy (RCM), optical coherence tomography, fluorescence imaging, high-frequency ultrasound and multispectral imaging, such as diffuse reflectance spectrophotometry imaging [23,24,25]. Some, such as RCM, have already proven to be useful in cost-benefit analyses and as an aid for dermatoscopic evaluation of atypical lesions [26,27]. These technologies can be successfully integrated into CAD with the use of machine learning. As a result, several CAD systems already employ spectroscopy and multispectral imaging [19].

Diffuse reflectance spectrophotometry (DRS) records light reflected off melanin, collagen, hemoglobin and other cutaneous chromophores [28], producing images of the visible and near-infrared spectrum (400–1000 nm) [23]. The light used can penetrate the papillary dermis, reaching 2 mm lesion depth. Examples of such systems include SIAscope and MelaFind. A recent Cochrane review put the sensitivity and specificity of multispectral imaging CAD systems at 0.929 (95% CI, 0.837 to 0.971) and 0.436 (95% CI, 0.248 to 0.645), respectively [23], with one RCT SIAscopy study in an unreferred population achieving a specificity of 0.725 with comparable sensitivities [29]. Combining spectrophotometry with other quantitative imaging modalities could yield even better diagnostic accuracy.

High-frequency ultrasound (HFUS) is an imaging modality using frequency of >20 MHz and is capable of diagnosing various skin lesions, including CM [30]. It is currently being used to measure tumor depth, predicting the required excision margins for the avoidance of biopsy before complete removal [31,32]. A Cochrane review of HFUS diagnostics of skin cancer was not able to perform a pooled analysis due to low study count and heterogeneity [33]. Qualitative markers of CM in two papers demonstrated sensitivities of 1.00 with specificities from 0.33 (95% CI, 0.20–0.48) to 0.73 (95% CI, 0.57–0.85). Two recorded attempts of quantitative measurements set at a sensitivity of 1.00 achieved a specificity of 0.93 (95% CI, 0.77–0.99) and 0.65 (95% CI, 0.51–0.76), respectively. However, all studies were deemed to be of low quality, with inherent biases [33]. A quantitative HFUS CM radiomics study evaluating parameters of tissue acoustics, texture and shape, managed to achieve accuracy of 0.824 [34]. Further CM ultrasound radiomics and CAD studies are lacking, and primarily focus on cancers of other sites [35,36,37,38].

In previous works, we tested the separate use of spectrophotometric analysis and HFUS on skin tumor depth prediction and quantitative differentiation of lesions (melanocytic naevus (MN) or CM) [28,34]. Moreover, we have performed an investigation by an indirect combination of a set of quantitative parameters estimated during the analysis of ultrasonic B-scan images and digital dermatoscopy images. The achieved probability of correct prediction utilizing the logistic regression model was 82%, with an AUROC of 0.908 [39].

In this study, we combine data gathered from optical dermatoscopy, spectrophotometric analysis (SIAscope) and HFUS to present a novel CAD system for the diagnosis of CM. The objective of this work is to increase the accuracy of the diagnosis of melanocytic skin tumors and CM by extracting sets of informative quantitative parameters from images of these three imaging technologies to train the classifiers. In order to shorten the computation time, combined sets of the most sensitive quantitative parameters extracted from the diagnostic images are used for the classification, instead of the whole images.

## 2. Description of the Proposed Technique

**Figure 1:**
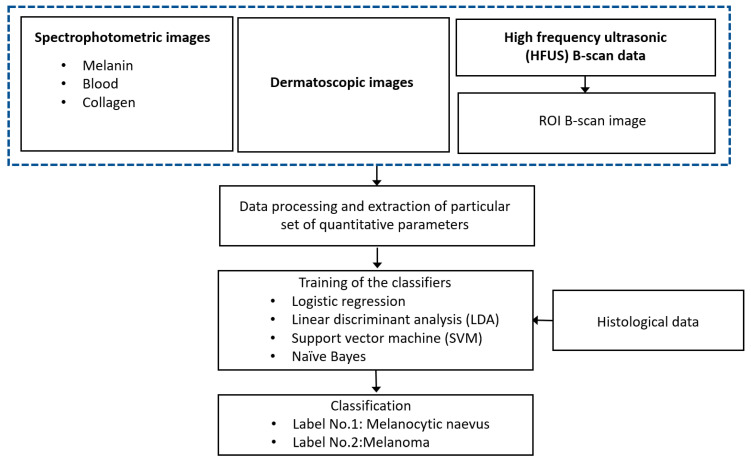
Flow chart description of the proposed diagnostic technique.

The flow chart describing the proposed diagnostic technique of CM is presented in Figure 1.

There are three sets of acquired spectrophotometric images (melanin, blood and collagen), one set of dermatoscopic images and HFUS B-scan images for groups of CM and melanocytic naevus (MN). The images were processed in Matlab 2020a (The MathWorks Inc., Natick, MA USA) by developing the special algorithms. In the case of ultrasonic B-scan, the region of interest (ROI) is extracted from the B-scan data by tracking the front and back surface reflections from the lesion boundaries. The sets of various quantitative parameters were extracted from different diagnostic images (dermatoscopic, spectrophotometric and ultrasonic) by processing these images for inputting to the classifiers. The four different algorithms for binary classification (CM or MN) using machine-learning techniques (logistic regression (LR), linear discriminant analysis (LDA), support vector machine (SVM) and Naive Bayes) were performed for the evaluation of diagnostic accuracy. The results of the automatic classification (accuracy, sensitivity, specificity, precision, Mathews correlation coefficient (MCC) and area under the ROC curve) are compared with the results of the histological examination. The proposed models could be used for reliable differentiation between the MN and CM.

## 3. Experimental Analysis and Clinical Measurements

In total, diagnostic images (dermatoscopic, spectrophotometric and ultrasonic B-scans) of skin lesions were acquired for 100 different patients. The age range of the examined patients was 17–87 years, an average 52.85 ± 17.33 years. According to the presence of artifacts within images, nine cases were excluded from the study. Therefore, further analysis was performed for 91 cases consisting of 50 naevi and 41 melanomas. All diagnoses were histopathologically confirmed by two experienced dermatopathologist. A third pathologist was called if there was discrepancy between the two pathologists. The range of MN thickness was from 0.2 mm up to 2.7 mm. The average thickness value of MN was 0.78 ± 0.54 mm, correspondingly, the range of CM thicknesses was from 0.22 mm up to 3.15 mm with an average value of 1.0 ± 0.7 mm.

Ultrasonic B-scan images were acquired by DUB-USB ultrasound imaging system (“Taberna pro medium”, Germany) possessing focussed transducer of 22 MHz central frequency. The sampling frequency was 100 MHz. The transducer was scanned up to 12.8 mm with a scanning step of 33 μm. The depth of the imaging region was 8 mm according to the velocity of ultrasound (1580 m/s) and length of signal acquisition window in time domain. Afterwards, the B-scan data were transferred to the computer for further processing.

Optical dermatoscopic and spectrophotometric images were acquired using spectrophotometer SimSys© (MedX Health Corp., Canada) operating in combined dermatoscopy-spectrophotometry modes and transferred to the computer for further analysis as well. The diameter of the imaging region was 11 mm. After surgical excision and during the routine histopathology, the diagnosis of skin lesions was confirmed.

All mentioned images were acquired at the Department of Skin and Venereal Diseases of Lithuanian University of Health Sciences. The presented study was approved by the regional ethics committee (No. P3-BE-2-25/2009, Date: 14 November 2017). The written informed consent was obtained from all patients before examination and surgical excision of the lesion. The diagnosis of skin lesions was confirmed by the routine histopathology after the surgical excision.

## 4. Data Processing

All acquired ultrasonic, dermatoscopic and spectrophotometric images were processed in Matlab R2020a. First of all, each ultrasonic A-scan signal of a B-scan image was interpolated achieving four times oversampling and reducing distortions of the digitized signal. The selection of front skin surface detection limits and maximum depth to analyze was performed manually for each B-scan data. The thickness of epidermis was selected as 0.1 mm throughout the data processing. It should be noted that typical skin tumors do not possess the expressed boundaries between tumors and the region of healthy tissues. That is why some false boundary points were detected. The second order low-pass Butterworth filter was used for the front and back surface contour follower. The optimal least-square polynomial approximation was performed for the detection of final boundaries. The minimal sum of differences between the polynomial and detected data points was used to select the optimal polynomial approximation that was in the range of 1st to 7th order. It can be expressed mathematically as follows [40]:

(1) $$D_{approx}=\sum_{m=1}^{M}\begin{array}{c} \left|\left(\sum_{n=0}^{N}a_{n}x{\left[m\right]}^{n}\right)-y\left[m\right]\right| \end{array}$$

where *D_approx_* is the sum of differences, *a_n_* and *y*[*m*] are the coefficients of a polynomial of degree *n* and *x*[*m*] is the polynomial term.

**Figure 2:**
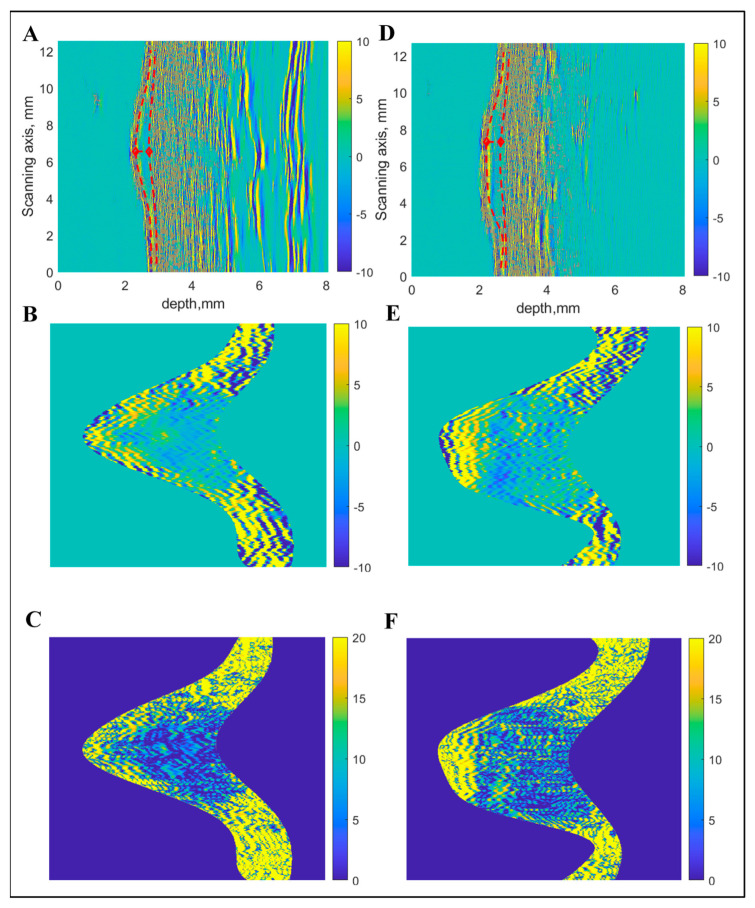
Boundary detection and extraction of region of interest from ultrasonic B-scan: Raw B-scan image (**A**), extracted region of interest with linear scale of amplitudes (**B**) and with logarithmic scale (**C**) in the case of melanocytic naevus (MN). Raw B-scan image (**D**), extracted region of interest with linear scale of amplitudes (**E**) and with logarithmic scale (**F**) in the case of cutaneous melanoma (CM).

In the next step, the average RMS value of the B-scan spectrum was calculated. In order to smooth the average RMS spectra, zero-phase digital filtering was applied. Thereafter, the front-surface reflections and reverberations (second-order reflections) were removed. In order to locate the back-surface reflections (bottom of CM), the same process was repeated, however, the thresholding was applied to data by calculating the moving average filter rectification (absolute values > 0.7). The thickness of CM/MN was considered as the maximum distance between the front and back surfaces. After extracting the region of interest (ROI) from each B-scan data, resulting regions were acquired and saved as images for further processing. The detection of boundaries and extracted images of interest are presented in Figure 2 in the case of both MN and CM. Afterwards, a quantitative analysis of the extracted images was carried out to characterize the spatial features. Overall, thirteen quantitative parameters were computed to provide input information for classification. After converting the image into grayscale, energy (Em) and entropy (Ep) was calculated from the histogram [41].

The range of energy value lies between 0 and 1, that is a numerical descriptor of uniformity. Entropy signifies the statistical measure of uncertainty and randomness and lies between 0 and log_2_(M) (M is the total number of levels in the histogram). The other quantitative parameters (mean, standard deviation, root mean square (RMS), variance, smoothness, skewness and kurtosis) of the intensity value distribution were computed in order to characterize the global distribution of intensity [42]. To characterize the texture feature of the image, the grayscale co-occurrence matrix (GSCM) was created from the image [43]. Afterwards, the four quantitative parameters (contrast, energy, correlation and homogeneity) from the GSCM were computed.

**Figure 3:**
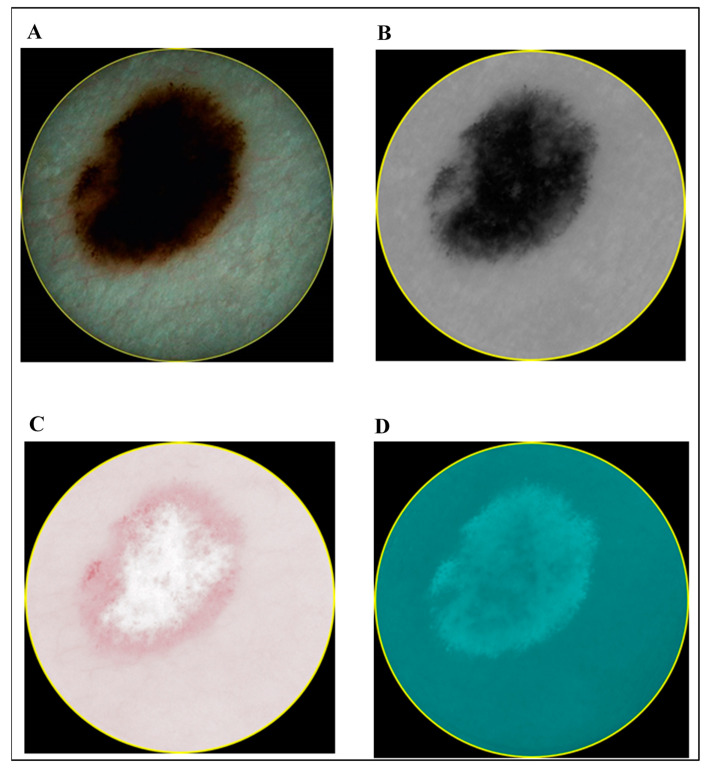
Images of cutaneous melanoma (CM): dermatoscopy (**A**) and different components acquired by spectrophotometry (melanin (**B**), blood (**C**) and collagen holes (**D**)).

**Figure 4:**
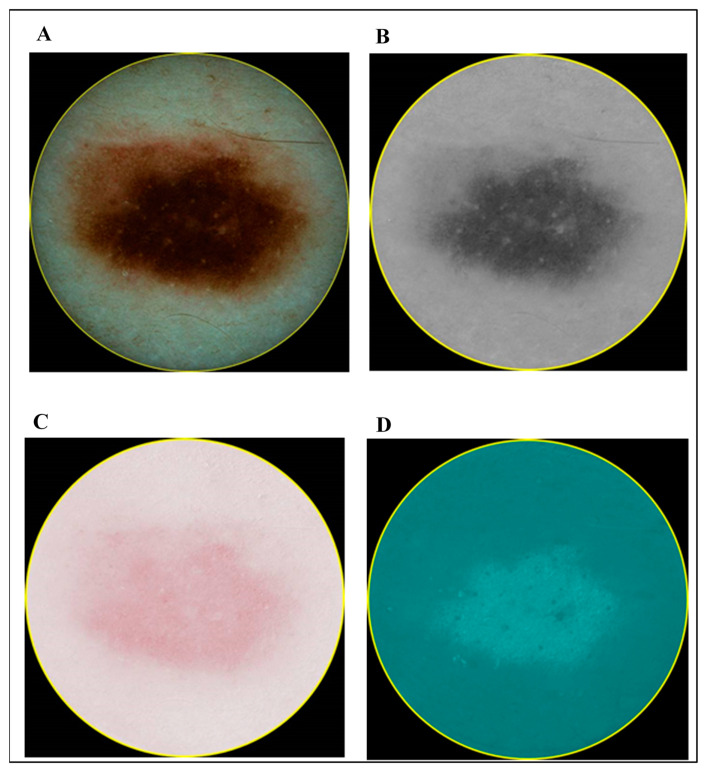
Images of melanocytic naevus (MN): dermatoscopy (**A**) and different components acquired by spectrophotometry (melanin (**B**), blood (**C**) and collagen (**D**)).

In contrast to the ultrasonic B-scan images, no initial preprocessing of dermatoscopic and spectrophotometric images was performed. The acquired images of CM and MN by dermatoscopy and spectrophotometry are shown in Figure 3 and Figure 4, respectively.

The same number (thirteen) of the quantitative parameters as computed in the case of ultrasonic B-scan images were computed from these images as well. However, not all of these parametric values were statistically significant to be used further for classification. Only those parameters that were statistically significant (*p* < 0.05 by using *t*-test) in each case (optical dermatoscopic images, spectrophotometric images and ultrasonic B-scan images), were selected to be used as input data for the binary classification algorithm. The selected sets of parameters are presented in Table 1.

## 5. Classification Algorithm

After computing the parameters from the mentioned imaging techniques, the next step was to train and evaluate the performance of classifiers. There are many classification methods (e.g., *K*—Nearest Neighbour, Decision Tree, Support Vector Machine, Artificial Neural Network (ANN), Neuro-Fuzzy, Fuzzy C-Mean (FCM), Naive Bayes and Clustering, linear regression etc.) that have been used in CAD of tissue affected by cancer [44,45].

In this work, four classification models (LR, LDA, Naive Bayes and SVM) have been used for binary classification (CM or MN) and their performances were compared.

One of the most common machine learning techniques for data classification is SVM [46]. The basic concept is based on the decision planes that separate the objects to differentiate the classes. If the data can be separated linearly, the simplest SVM is linear. If the data cannot be separated linearly, kernel SVM with radial base function (RBF) can be utilized [47] to classify the data into CM or MN. In our case, SVM with the utilization of RBF kernel is used. In the logistic regression model, the predictor variables can be scale-dependent and quantitative, however, the dependent variable lies in membership or non-membership category [48]. The mechanism on which logistic regression works is called Logit. In comparison to the multiple regression, logistic regression requires less assumption and hence, it is more flexible. Another classification model used in this work is Linear discriminant analysis (LDA) or Fischer discriminants which is a common technique for dimensionality reduction and classification [49]. The method aims to maximize the ratio of the between-group variance and the within-group variance [50]. The Naive Bayes classifiers simple probabilistic classifiers that are based on the application of Bayes′ theorem with strong distinguished assumptions between the features [51,52]. As Naive Bayes classifier is highly scalable, it requires the linear parameters for learning. During the training stage, the 10-fold cross-validation process was used to build all classifier models for the computation of optimized parameters.

The standard parameters were computed to measure the performance of these four models for binary classification. The five threshold-dependent parameters (sensitivity (***S_e_***), specificity (***S_p_***), accuracy (***A_c_***) and Matthews correlation coefficient (MCC)) and one threshold-independent parameter (area under ROC (AUROC)) were employed to measure the performance.

The threshold dependent parameters can be expressed by following mathematical equations [53]:

(2) $$S_{e}=\frac{TP}{TP+FN}*100$$

(3) $$S_{p}=\frac{TN}{TN+FP}*100$$

(4) $$A_{c}=\frac{TP+TN}{TP+FP+TN+FN}*100$$

(5) $$P_{r}=\frac{TP}{TP+FP}*100$$

(6) $$MCC=\frac{\left(TP\cdot TN\right)+\left(FP\cdot FN\right)}{\sqrt{\left(TP+FP\right)\cdot \left(TP+FN\right)\cdot \left(TN+FP\right)\cdot \left(TN+FN\right)}}$$

where *S_e_*, *S_p_*, *A_c_*, *P_r_* and *MCC* denote the sensitivity, specificity, accuracy, precision and Matthews correlation coefficient (MCC), respectively, and *FP*, *FN*, *TP* and *TN* denote the false positive, false negative, true positive and true negative, respectively.

The standard AUROC curve was generated by plotting the sensitivity against the false positive rate at different thresholds. Afterwards, the area under the ROC curve was estimated to evaluate the AUROC parameter.

## 6. Results and Discussion

The performance of combining all three imaging techniques (dermatoscopy, spectrophotometry and ultrasound) based on the sets of quantitative parameters extracted from different images provided by particular imaging technique as discussed in Section 4 is compared. The results are sequentially discussed in this Section to show the improvement with the combination of all three imaging techniques compared to the combination of any two imaging techniques.

Case 1: Combining Quantitative Parameters from Dermatoscopic and Spectrophotometric Images

First of all, the classification models are developed by combining only the quantitative parameters computed from the dermatoscopy and spectrophotometry (melanin, blood and collagen) techniques. The results of the classifiers are presented in Table 2. The highest accuracy (90.11%), sensitivity (85.37%), specificity (94.00%), precision (92.11%), MCC (0.801) and AUROC (0.972) were achieved with SVM in comparison to all other models. Moreover, the accuracy was more than 74% for all classifiers.

Case 2: Combining Quantitative Parameters from Dermatoscopic and Ultrasonic B-scan Images

In the next step, the classification models were developed by combining the quantitative parameters of dermatoscopic and ultrasonic B-scan images. The classification results with this combination have been presented in Table 3. The performance of SVM (Table 3) was better in this case as compared to the previous combination (case 1, Table 2) by considering all statistical parameters except the AUROC. Moreover, the SVM model again showed the highest accuracy (91.21%), sensitivity (80.49%), specificity (100%), precision (100%), MCC (0.833) and AUROC (0.961) among all models. Moreover, the accuracy was more than 76% for all classifiers. The performance of Naive Bayes model (accuracy and MCC) was also improved as compared to the previous combination (case 1, Table 2). However, the performance of LR and LDA (Table 3) was reduced in comparison to a combination of spectrometric and optical dermatoscopic imaging techniques (case 1, Table 2).

Case 3: Combination of Spectrophotometry and HFUS Imaging Techniques

In this case, the quantitative parameters (Table 4) of both spectrophotometric and ultrasonic B-scan images are utilized in order to develop the classification models. The results of classifiers are presented in Table 4. More than 85% accuracy and sensitivity was achieved for LR, LDA and SVM. By considering all the statistical parameters obtained from the classifiers, the SVM shows the highest performance in this case with an accuracy of 95.60% and MCC of 0.912. As shown in Table 4, the performance of SVM was better in comparison to the case 1 (Table 2) and case 2 (Table 3). The AUROC for all classifiers is also higher in this case as comparison to the case 2 and case 3, except for LDA for which AUROC (0.905) is slightly less than case 1 (0.906). The performance of Naive Bayes remains the lowest.

Case 4: Combination of All Three (Optical Dermatoscopy, Spectrophotometry and HFUS) Imaging Techniques

Finally, the quantitative parameters (Table 1) of all three different types of images (i.e., optical dermatoscopic, spectrophotometric and ultrasonic B-scan) are utilized in classification and presented in Table 5. In comparison to all the three cases mentioned above, the higher accuracy of more than 90% was achieved by using three classification models (LR, LDA and SVM). Moreover, the MCC and AUROC for all classifiers were highest in this case as compared to the previous three cases. The higher values of other parameters (sensitivity, specificity and precision) also signify the improvement of performance by combining three imaging techniques instead of the combination of any two of them.

It is clearly observed that SVM outperformed all other classifiers; on another hand, Naive Bayes showed the worst performance. SVM is proven to be the optimal for linearly separable cases and its strategy to determine maximum-margin hyperplane is one of the best to reduce the prediction error [54]. In general, SVM is better for a two-class classification problem with a smaller number of features [55,56]. However, Naive Bayes can handle more features easily. Although the Naive Bayesian classification is an effective model for diagnosis melanoma, the decision tree algorithm is not well suited in this domain [57]. It is important to consider that SVM is not so popular with large data sets as it requires a significant amount of training time; however, in our research, this was not the case [56]. In comparison to the latest research work on SVM classification for melanoma (85.19% [58], 92.1% [59], 96% [60], 90% [61], 97.32% [62]), we achieved 98.9% accuracy with the proposed technique.

## 7. Conclusions

In our study a novel diagnostic system by combining the three different non-invasive medical imaging techniques (optical dermatoscopy, spectrophotometry and high-frequency ultrasound) is proposed for the reliable differentiation of CM and MN. In the case of having a limited number of diagnostic images and in order to expedite the processing, the sets of most sensitive quantitative parameters from the images were acquired and used as input of classifiers instead of images themselves. The binary classification results, combining the three imaging techniques, showed the highest accuracy of more than 90% for LR, LDA and SVM classifiers, which is not possible to achieve by combining only two imaging techniques. The obtained results reveal that SVM is the most suitable classification model for the detection of CM with accuracy of 98.9%, MCC of 0.978, sensitivity of 97.5%, AUROC of 0.999 and specificity and precision of 100%. The second classification model according to achieved high accuracy of 92.3% was LR. The proposed clinical decision support system can supplement non-invasive diagnostic methods already existing in clinical practice. Furthermore, big data analysis and deep learning neural networks (e.g., convolutional neural networks) could be used to implement a more accurate diagnostic system by using this approach in the future, after acquiring the required higher number of diagnostic images by aforementioned different imaging techniques.

## Figures and Tables

**Table 1:** Numbers of selected quantitative parameters to be used for binary classification of images acquired using different imaging technologies (1—entropy, 2—energy, 3—contrast, 4—correlation, 5—energy from GSCM, 6—homogeneity, 7—mean, 8—standard deviation, 9—RMS, 10—variance, 11—smoothness, 12—kurtosis, 13—skewness) (x denotes the statistically significant (*p* < 0.05) parameter).

Type of Imaging Technology and Images	Numbers of Selected Quantitative Parameters (*p* < 0.05) to be Used for Classification												
	1	2	3	4	5	6	7	8	9	10	11	12	13
Optical dermatoscopy		x	x			x			x				
Spectrophotometry (melanin component)	x						x		x				
Spectrophotometry (blood component)	x		x	x	x	x						x	x
Spectrophotometry (collagen component)	x							x		x		x	
Ultrasonic B-scan					x	x	x	x		x		x	x

**Table 2:** Performance of classifiers by combining optical dermatoscopy and spectrophotometry imaging techniques to classify cutaneous melanoma (CM) and melanocytic naevus (MN).

Type of Classifier	Statistical Parameters					
	Accuracy, %	Sensitivity, %	Specificity, %	Precision, %	Matthews Correlation Coefficient (MCC)	Area under the ROC Curve
Logistic regression (LR)	89.01	85.37	92.00	89.74	0.778	0.918
Linear discriminant analysis (LDA)	84.62	78.05	90.00	86.49	0.689	0.906
Support vector machine (SVM)	90.11	85.37	94.0	92.11	0.801	0.972
Naive Bayes	74.73	58.54	88.00	80.00	0.493	0.813

**Table 3:** Performance of classifiers by combining optical dermatoscopy and high-frequency ultrasonography (HFUS) imaging techniques.

Type of Classifier	Statistical Parameters					
	Accuracy, %	Sensitivity, %	Specificity, %	Precision, %	Matthews Correlation Coefficient (MCC)	Area under the ROC Curve
Logistic regression (LR)	82.42	78.05	86.00	82.05	0.644	0.908
Linear discriminant analysis (LDA)	80.22	73.17	86.00	81.08	0.599	0.906
Support vector machine (SVM)	91.21	80.49	100	100	0.833	0.961
Naive Bayes	76.92	75.61	78.00	73.81	0.535	0.812

**Table 4:** Performance of classifiers by combining spectrophotometry and HFUS imaging techniques.

Type of Classifier	Statistical Parameters					
	Accuracy, %	Sensitivity, %	Specificity, %	Precision, %	Matthews Correlation Coefficient (MCC)	Area under the ROC Curve
Logistic regression (LR)	85.71	85.37	86.00	83.33	0.712	0.928
Linear discriminant analysis (LDA)	86.81	85.37	88.00	85.37	0.734	0.905
Support vector machine (SVM)	95.60	92.68	98.00	97.44	0.912	0.996
Naive Bayes	73.63	65.85	80.00	72.97	0.465	0.82

**Table 5:** Performance of classifiers by combining optical dermatoscopy, spectrophotometry (melanin, blood and collagen) and HFUS imaging techniques.

Type of Classifier	Statistical Parameters					
	Accuracy, %	Sensitivity, %	Specificity, %	Precision, %	Matthews Correlation Coefficient (MCC)	Area under the ROC Curve
Logistic regression (LR)	92.31	87.80	96.00	94.74	0.846	0.956
Linear discriminant analysis (LDA)	90.11	85.37	94.00	92.11	0.801	0.939
Support vector machine (SVM)	98.9	97.56	100	100	0.978	0.999
Naive Bayes	75.82	65.85	84.00	77.14	0.51	0.829

## References

[1] Ferlay J., Colombet M., Soerjomataram I., Dyba T., Randi G., Bettio M., Gavin A., Visser O., Bray F. (2018). Cancer incidence and mortality patterns in Europe: Estimates for 40 countries and 25 major cancers in 2018. Eur. J. Cancer.

[2] Ferlay J., Colombet M., Soerjomataram I., Mathers C., Parkin D.M., Piñeros M., Znaor A., Bray F. (2019). Estimating the global cancer incidence and mortality in 2018: GLOBOCAN sources and methods. Int. J. Cancer.

[3] Force U.P.S.T., Bibbins-Domingo K., Grossman D.C., Curry S.J., Davidson K.W., Ebell M., Epling J.W., García F.A.R., Gillman M.W., Kemper A.R. (2016). Screening for Skin Cancer. JAMA.

[4] Gardner L.J., Strunck J.L., Wu Y.P., Grossman D. (2019). Current controversies in early-stage melanoma: Questions on incidence, screening, and histologic regression. J. Am. Acad. Dermatol..

[5] Watts C.G., Dieng M., Morton R.L., Mann G.J., Menzies S.W., Cust A.E. (2014). Clinical practice guidelines for identification, screening and follow-up of individuals at high risk of primary cutaneous melanoma: A systematic review. Br. J. Dermatol..

[6] Gershenwald J.E., Scolyer R.A., Hess K.R., Sondak V.K., Long G.V., Ross M.I., Lazar A.J., Faries M.B., Kirkwood J.M., McArthur G.A. (2017). For members of the American Joint Committee on Cancer Melanoma Expert Panel and the International Melanoma Database and Discovery Platform Melanoma staging: Evidence-based changes in the American Joint Committee on Cancer eighth edition cancer staging manual. CA Cancer J. Clin..

[7] Schadendorf D., van Akkooi A.C.J., Berking C., Griewank K.G., Gutzmer R., Hauschild A., Stang A., Roesch A., Ugurel S. (2018). Melanoma. Lancet.

[8] Amin M.B., Greene F.L., Edge S.B., Compton C.C., Gershenwald J.E., Brookland R.K., Meyer L., Gress D.M., Byrd D.R., Winchester D.P. (2017). The Eighth Edition AJCC Cancer Staging Manual: Continuing to build a bridge from a population-based to a more “personalized” approach to cancer staging. CA Cancer J. Clin..

[9] Swetter S.M., Tsao H., Bichakjian C.K., Curiel-Lewandrowski C., Elder D.E., Gershenwald J.E., Guild V., Grant-Kels J.M., Halpern A.C., Johnson T.M. (2019). Guidelines of care for the management of primary cutaneous melanoma. J. Am. Acad. Dermatol..

[10] Vestergaard M.E., Macaskill P., Holt P.E., Menzies S.W. (2008). Dermoscopy compared with naked eye examination for the diagnosis of primary melanoma: A meta-analysis of studies performed in a clinical setting. Br. J. Dermatol..

[11] Salerni G., Terán T., Puig S., Malvehy J., Zalaudek I., Argenziano G., Kittler H. (2012). Meta-analysis of digital dermoscopy follow-up of melanocytic skin lesions: A study on behalf of the International Dermoscopy Society. J. Eur. Acad. Dermatol. Venereol..

[12] Rabinowitz A., Silvers D. (1996). Dermatopathology standards. J. Cutan. Pathol..

[13] Farmer E.R., Gonin R., Hanna M.P. (1996). Discordance in the histopathologic diagnosis of melanoma and melanocytic nevi between expert pathologists. Hum. Pathol..

[14] Elmore J.G., Barnhill R.L., Elder D.E., Longton G.M., Pepe M.S., Reisch L.M., Carney P.A., Titus L.J., Nelson H.D., Onega T. (2017). Pathologists’ diagnosis of invasive melanoma and melanocytic proliferations: Observer accuracy and reproducibility study. BMJ.

[15] Hekler A., Utikal J.S., Enk A.H., Berking C., Klode J., Schadendorf D., Jansen P., Franklin C., Holland-Letz T., Krahl D. (2019). Pathologist-level classification of histopathological melanoma images with deep neural networks. Eur. J. Cancer.

[16] Lambin P., Leijenaar R.T.H., Deist T.M., Peerlings J., de Jong E.E.C., van Timmeren J., Sanduleanu S., Larue R.T.H.M., Even A.J.G., Jochems. A. (2017). Radiomics: The bridge between medical imaging and personalized medicine. Nat. Rev. Clin. Oncol..

[17] Esteva A., Kuprel B., Novoa R.A., Ko J., Swetter S.M., Blau H.M., Thrun S. (2017). Dermatologist-level classification of skin cancer with deep neural networks. Nature.

[18] Brinker T.J., Hekler A., Enk A.H., Klode J., Hauschild A., Berking C., Schilling B., Haferkamp S., Schadendorf D., Fröhling S. (2019). Collaborators A convolutional neural network trained with dermoscopic images performed on par with 145 dermatologists in a clinical melanoma image classification task. Eur. J. Cancer.

[19] Dick V., Sinz C., Mittlböck M., Kittler H., Tschandl P. (2019). Accuracy of Computer-Aided Diagnosis of Melanoma: A Meta-analysis. JAMA Dermatol..

[20] Haenssle H.A., Fink C., Schneiderbauer R., Toberer F., Buhl T., Blum A., Kalloo A., Hassen A.B.H., Thomas L., Enk A. (2018). Man against machine: Diagnostic performance of a deep learning convolutional neural network for dermoscopic melanoma recognition in comparison to 58 dermatologists. Ann. Oncol..

[21] Cui X., Wei R., Gong L., Qi R., Zhao Z., Chen H., Song K., Abdulrahman A.A.A., Wang Y., Chen J.Z.S. (2019). Assessing the effectiveness of artificial intelligence methods for melanoma: A retrospective review. J. Am. Acad. Dermatol..

[22] Brinker T.J., Hekler A., Enk A.H., Klode J., Hauschild A., Berking C., Schilling B., Haferkamp S., Schadendorf D., Holland-Letz T. (2019). Collaborators Deep learning outperformed 136 of 157 dermatologists in a head-to-head dermoscopic melanoma image classification task. Eur. J. Cancer.

[23] Ferrante di Ruffano L., Takwoingi Y., Dinnes J., Chuchu N., Bayliss S.E., Davenport C., Matin R.N., Godfrey K., O’Sullivan C., Gulati A. (2018). Cochrane Skin Cancer Diagnostic Test Accuracy Group Computer-assisted diagnosis techniques (dermoscopy and spectroscopy-based) for diagnosing skin cancer in adults. Cochrane Database Syst. Rev..

[24] Schneider S.L., Kohli I., Hamzavi I.H., Council M.L., Rossi A.M., Ozog D.M. (2019). Emerging imaging technologies in dermatology: Part I: Basic principles. J. Am. Acad. Dermatol..

[25] Schneider S.L., Kohli I., Hamzavi I.H., Council M.L., Rossi A.M., Ozog D.M. (2019). Emerging imaging technologies in dermatology: Part II: Applications and limitations. J. Am. Acad. Dermatol..

[26] Pellacani G., Witkowski A., Cesinaro A.M., Losi A., Colombo G.L., Campagna A., Longo C., Piana S., de Carvalho N., Giusti F. (2016). Cost-benefit of reflectance confocal microscopy in the diagnostic performance of melanoma. J. Eur. Acad. Dermatol. Venereol..

[27] Mataca E., Migaldi M., Cesinaro A.M. (2018). Impact of Dermoscopy and Reflectance Confocal Microscopy on the Histopathologic Diagnosis of Lentigo Maligna/Lentigo Maligna Melanoma. Am. J. Dermatopathol..

[28] Sakalauskienė K., Valiukevičienė S., Raišutis R., Linkevičiūtė G. (2018). The significance of spectrophotometric image analysis for diagnosis of the melanocytic skin tumours in association with their thickness. Skin Res. Technol..

[29] Walter F.M., Morris H.C., Humphrys E., Hall P.N., Prevost A.T., Burrows N., Bradshaw L., Wilson E.C.F., Norris P., Walls J. (2012). Effect of adding a diagnostic aid to best practice to manage suspicious pigmented lesions in primary care: Randomised controlled trial. BMJ.

[30] Kleinerman R., Whang T.B., Bard R.L., Marmur E.S. (2012). Ultrasound in dermatology: Principles and applications. J. Am. Acad. Dermatol..

[31] Jasaitiene D., Valiukeviciene S., Linkeviciute G., Raisutis R., Jasiuniene E., Kazys R. (2011). Principles of high-frequency ultrasonography for investigation of skin pathology. J. Eur. Acad. Dermatol. Venereol..

[32] Chaput L., Laurent E., Pare A., Sallot A., Mourtada Y., Ossant F., Vaillant L., Patat F., Machet L. (2018). One-Step surgical removal of cutaneous melanoma with surgical margins based on preoperative ultrasound measurement of the thickness of the melanoma. Eur. J. Dermatol..

[33] Dinnes J., Bamber J., Chuchu N., Bayliss S.E., Takwoingi Y., Davenport C., Godfrey K., O’Sullivan C., Matin R.N., Deeks J.J. (2018). Cochrane Skin Cancer Diagnostic Test Accuracy Group High-frequency ultrasound for diagnosing skin cancer in adults. Cochrane Database Syst. Rev..

[34] Andrėkutė K., Linkevičiūtė G., Raišutis R., Valiukevičienė S., Makštienė J. (2016). Automatic Differential Diagnosis of Melanocytic Skin Tumors Using Ultrasound Data. Ultrasound Med. Biol..

[35] Larue R.T., Defraene G., de Ruysscher D., Lambin P., van Elmpt W. (2017). Quantitative radiomics studies for tissue characterization: A review of technology and methodological procedures. Br. J. Radiol..

[36] Yao Z., Dong Y., Wu G., Zhang Q., Yang D., Yu J.H., Wang W.P. (2018). Preoperative diagnosis and prediction of hepatocellular carcinoma: Radiomics analysis based on multi-modal ultrasound images. BMC Cancer.

[37] Guo Y., Hu Y., Qiao M., Wang Y., Yu J., Li J., Chang C. (2018). Radiomics Analysis on Ultrasound for Prediction of Biologic Behavior in Breast Invasive Ductal Carcinoma. Clin. Breast Cancer.

[38] Hu H., Wang Z., Huang X., Chen S., Zheng X., Ruan S., Xie X., Lu M., Yu J., Tian J. (2019). Ultrasound-based radiomics score: A potential biomarker for the prediction of microvascular invasion in hepatocellular carcinoma. Eur. Radiol..

[39] Drulyte I., Ruzgas T., Raisutis R., Valiukeviciene S., Linkeviciute G. (2018). Application of automatic statistical post-processing method for analysis of ultrasonic and digital dermatoscopy images. Libyan J. Med..

[40] Andrekute K., Valiukeviciene S., Raisutis R., Linkeviciute G., Makstiene J., Kliunkiene R. (2016). Automated Estimation of Melanocytic Skin Tumor Thickness by Ultrasonic Radiofrequency Data. J. Ultrasound Med..

[41] Rey-Barroso L., Burgos-Fernández F.J., Delpueyo X., Ares M., Royo S., Malvehy J., Puig S., Vilaseca M. (2018). Visible and Extended Near-Infrared Multispectral Imaging for Skin Cancer Diagnosis. Sensors.

[42] Echegaray S., Bakr S., Rubin D.L., Napel S. (2018). Quantitative Image Feature Engine (QIFE): An Open-Source, Modular Engine for 3D Quantitative Feature Extraction from Volumetric Medical Images. J. Digit. Imaging.

[43] Haralick R.M., Shanmugam K., Dinstein I. (1973). Textural Features for Image Classification. IEEE Trans. Syst. Man Cybern..

[44] Kotsiantis S.B., Zaharakis I.D., Pintelas P.E. (2006). Machine learning: A review of classification and combining techniques. Artif. Intell. Rev..

[45] Shi Z., He L., Suzuki K., Nakamura T., Itoh H. (2009). Survey on Neural Networks Used for Medical Image Processing. Int. J. Comput. Sci..

[46] Pisner D.A., Schnyer D.M., Mechelli A., Vieira S. (2020). Chapter 6-Support vector machine. Machine Learning.

[47] Hiam A., Eman S., Michael O.J., Mufeed M. Enhancement of 3D modeling and classification of microcalcifications in breast computed tomography (BCT). Proceedings of the Society of Photo-Optical Instrumentation Engineers (SPIE) Conference Series.

[48] Whiteman D.C., Thompson B.S., Thrift A.P., Hughes M., Muranushi C., Neale R.E., Green A.C., Olsen C.M., Whiteman D.C., Green A.C. (2016). A Model to Predict the Risk of Keratinocyte Carcinomas. J. Investig. Dermatol..

[49] Mohanty N., John A.L., Manmatha R., Rath T.M. (2013). Chapter 10-Shape-Based Image Classification and Retrieval. Handb. Stat..

[50] Liu C. (2014). Discriminant analysis and similarity measure. Pattern Recognit..

[51] Jadhav P., Guru S.K. (2017). Image classification using naive bayes model for deep head pose estimation. Int. J. Adv. Eng. Res. Dev..

[52] Jiang L., Zhang H., Cai Z. (2009). A Novel Bayes Model: Hidden Naive Bayes. IEEE Trans. Knowl. Data Eng..

[53] Bhalla S., Kaur H., Dhall A., Raghava G.P.S. (2019). Prediction and Analysis of Skin Cancer Progression using Genomics Profiles of Patients. Sci. Rep..

[54] Niranjan M. Support Vector Machines: A Tutorial Overview and Critical Appraisal. Proceedings of the IEE Colloquium on Applied Statistical Pattern Recognition 1999.

[55] Murugan A., Nair S.A., Kumar K.P.S. (2019). Detection of Skin Cancer Using SVM, Random Forest and kNN Classifiers. J. Med. Syst..

[56] Cervantes J., Garcia-Lamont F., Rodríguez-Mazahua L., Lopez A. (2020). A comprehensive survey on support vector machine classification: Applications, challenges and trends. Neurocomputing.

[57] Arasi M.A., Dahshan E., Horbaty S.M., Salem A.M. (2016). Malignant Melanoma Detection Based on Machine Learning Techniques: A Survey. Egypt. Comput. Sci. J..

[58] Seeja R.D., Suresh A. (2019). Deep Learning Based Skin Lesion Segmentation and Classification of Melanoma Using Support Vector Machine (SVM). Asian Pac. J. Cancer Prev..

[59] Alquran H., Abu Qasmieh I., Alqudah A.M., Alhammouri S., Alawneh E., AbuGhazaleh A., Hasayen F. (2017). The melanoma skin cancer detection and classification using support vector machine. Proceedings of the 2017 IEEE Jordan Conference on Applied Electrical Engineering and Computing Technologies (AEECT).

[60] Patil M., Dongre N. (2020). Melanoma Detection Using HSV with SVM Classifier and De-duplication Technique to Increase Efficiency. Commun. Comput. Inf. Sci..

[61] Jaworek-Korjakowska J. (2016). Computer-Aided Diagnosis of Micro-Malignant Melanoma Lesions Applying Support Vector Machines. BioMed Res. Int..

[62] Bakheet S. (2017). An SVM Framework for Malignant Melanoma Detection Based on Optimized HOG Features. Computation.

